# A volar skin excisional wound model for *in situ* evaluation of multiple-appendage regeneration and innervation

**DOI:** 10.1093/burnst/tkad027

**Published:** 2023-06-29

**Authors:** Huanhuan Gao, Yiqiong Liu, Ziwei Shi, Hongliang Zhang, Mengyang Wang, Huating Chen, Yan Li, Shaifei Ji, Jiangbing Xiang, Wei Pi, Laixian Zhou, Yiyue Hong, Lu Wu, Aizhen Cai, Xiaobing Fu, Xiaoyan Sun

**Affiliations:** Research Center for Tissue Repair and Regeneration Affiliated to Medical Innovation Research Department and 4^th^ Medical Center, PLA General Hospital and PLA Medical College; PLA Key Laboratory of Tissue Repair and Regenerative Medicine and Beijing Key Research Laboratory of Skin Injury, Repair and Regeneration; Research Unit of Trauma Care, Tissue Repair and Regeneration, Chinese Academy of Medical Sciences, 2019RU051, Beijing 100048, P. R. China; Research Center for Tissue Repair and Regeneration Affiliated to Medical Innovation Research Department and 4^th^ Medical Center, PLA General Hospital and PLA Medical College; PLA Key Laboratory of Tissue Repair and Regenerative Medicine and Beijing Key Research Laboratory of Skin Injury, Repair and Regeneration; Research Unit of Trauma Care, Tissue Repair and Regeneration, Chinese Academy of Medical Sciences, 2019RU051, Beijing 100048, P. R. China; Institute of Chemistry, Chinese Academy of Sciences, 2 Beiyi Street, Zhong guan cun, Beijing 100190, P. R. China; Research Center for Tissue Repair and Regeneration Affiliated to Medical Innovation Research Department and 4^th^ Medical Center, PLA General Hospital and PLA Medical College; PLA Key Laboratory of Tissue Repair and Regenerative Medicine and Beijing Key Research Laboratory of Skin Injury, Repair and Regeneration; Research Unit of Trauma Care, Tissue Repair and Regeneration, Chinese Academy of Medical Sciences, 2019RU051, Beijing 100048, P. R. China; Research Center for Tissue Repair and Regeneration Affiliated to Medical Innovation Research Department and 4^th^ Medical Center, PLA General Hospital and PLA Medical College; PLA Key Laboratory of Tissue Repair and Regenerative Medicine and Beijing Key Research Laboratory of Skin Injury, Repair and Regeneration; Research Unit of Trauma Care, Tissue Repair and Regeneration, Chinese Academy of Medical Sciences, 2019RU051, Beijing 100048, P. R. China; Research Center for Tissue Repair and Regeneration Affiliated to Medical Innovation Research Department and 4^th^ Medical Center, PLA General Hospital and PLA Medical College; PLA Key Laboratory of Tissue Repair and Regenerative Medicine and Beijing Key Research Laboratory of Skin Injury, Repair and Regeneration; Research Unit of Trauma Care, Tissue Repair and Regeneration, Chinese Academy of Medical Sciences, 2019RU051, Beijing 100048, P. R. China; Research Center for Tissue Repair and Regeneration Affiliated to Medical Innovation Research Department and 4^th^ Medical Center, PLA General Hospital and PLA Medical College; PLA Key Laboratory of Tissue Repair and Regenerative Medicine and Beijing Key Research Laboratory of Skin Injury, Repair and Regeneration; Research Unit of Trauma Care, Tissue Repair and Regeneration, Chinese Academy of Medical Sciences, 2019RU051, Beijing 100048, P. R. China; Research Center for Tissue Repair and Regeneration Affiliated to Medical Innovation Research Department and 4^th^ Medical Center, PLA General Hospital and PLA Medical College; PLA Key Laboratory of Tissue Repair and Regenerative Medicine and Beijing Key Research Laboratory of Skin Injury, Repair and Regeneration; Research Unit of Trauma Care, Tissue Repair and Regeneration, Chinese Academy of Medical Sciences, 2019RU051, Beijing 100048, P. R. China; Research Center for Tissue Repair and Regeneration Affiliated to Medical Innovation Research Department and 4^th^ Medical Center, PLA General Hospital and PLA Medical College; PLA Key Laboratory of Tissue Repair and Regenerative Medicine and Beijing Key Research Laboratory of Skin Injury, Repair and Regeneration; Research Unit of Trauma Care, Tissue Repair and Regeneration, Chinese Academy of Medical Sciences, 2019RU051, Beijing 100048, P. R. China; Research Center for Tissue Repair and Regeneration Affiliated to Medical Innovation Research Department and 4^th^ Medical Center, PLA General Hospital and PLA Medical College; PLA Key Laboratory of Tissue Repair and Regenerative Medicine and Beijing Key Research Laboratory of Skin Injury, Repair and Regeneration; Research Unit of Trauma Care, Tissue Repair and Regeneration, Chinese Academy of Medical Sciences, 2019RU051, Beijing 100048, P. R. China; Research Center for Tissue Repair and Regeneration Affiliated to Medical Innovation Research Department and 4^th^ Medical Center, PLA General Hospital and PLA Medical College; PLA Key Laboratory of Tissue Repair and Regenerative Medicine and Beijing Key Research Laboratory of Skin Injury, Repair and Regeneration; Research Unit of Trauma Care, Tissue Repair and Regeneration, Chinese Academy of Medical Sciences, 2019RU051, Beijing 100048, P. R. China; Research Center for Tissue Repair and Regeneration Affiliated to Medical Innovation Research Department and 4^th^ Medical Center, PLA General Hospital and PLA Medical College; PLA Key Laboratory of Tissue Repair and Regenerative Medicine and Beijing Key Research Laboratory of Skin Injury, Repair and Regeneration; Research Unit of Trauma Care, Tissue Repair and Regeneration, Chinese Academy of Medical Sciences, 2019RU051, Beijing 100048, P. R. China; Research Center for Tissue Repair and Regeneration Affiliated to Medical Innovation Research Department and 4^th^ Medical Center, PLA General Hospital and PLA Medical College; PLA Key Laboratory of Tissue Repair and Regenerative Medicine and Beijing Key Research Laboratory of Skin Injury, Repair and Regeneration; Research Unit of Trauma Care, Tissue Repair and Regeneration, Chinese Academy of Medical Sciences, 2019RU051, Beijing 100048, P. R. China; Research Institute of General Surgery, Department of General Surgery, the First Medical Center, PLA General Hospital, 28 Fu Xing Road, Beijing 100853, P. R. China; Research Center for Tissue Repair and Regeneration Affiliated to Medical Innovation Research Department and 4^th^ Medical Center, PLA General Hospital and PLA Medical College; PLA Key Laboratory of Tissue Repair and Regenerative Medicine and Beijing Key Research Laboratory of Skin Injury, Repair and Regeneration; Research Unit of Trauma Care, Tissue Repair and Regeneration, Chinese Academy of Medical Sciences, 2019RU051, Beijing 100048, P. R. China; Research Center for Tissue Repair and Regeneration Affiliated to Medical Innovation Research Department and 4^th^ Medical Center, PLA General Hospital and PLA Medical College; PLA Key Laboratory of Tissue Repair and Regenerative Medicine and Beijing Key Research Laboratory of Skin Injury, Repair and Regeneration; Research Unit of Trauma Care, Tissue Repair and Regeneration, Chinese Academy of Medical Sciences, 2019RU051, Beijing 100048, P. R. China

**Keywords:** Wound healing, Animal model, Multiple appendages, Innervation, Regeneration evaluation, Hair follicles, Sweat gland, Sebaceous gland

## Abstract

**Background:**

Promoting rapid wound healing with functional recovery of all skin appendages is the main goal of regenerative medicine. So far current methodologies, including the commonly used back excisional wound model (BEWM) and paw skin scald wound model, are focused on assessing the regeneration of either hair follicles (HFs) or sweat glands (SwGs). How to achieve *de novo* appendage regeneration by synchronized evaluation of HFs, SwGs and sebaceous glands (SeGs) is still challenging. Here, we developed a volar skin excisional wound model (VEWM) that is suitable for examining cutaneous wound healing with multiple-appendage restoration, as well as innervation, providing a new research paradigm for the perfect regeneration of skin wounds.

**Methods:**

Macroscopic observation, iodine–starch test, morphological staining and qRT-PCR analysis were used to detect the existence of HFs, SwGs, SeGs and distribution of nerve fibres in the volar skin. Wound healing process monitoring, HE/Masson staining, fractal analysis and behavioral response assessment were performed to verify that VEWM could mimic the pathological process and outcomes of human scar formation and sensory function impairment.

**Results:**

HFs are limited to the inter-footpads. SwGs are densely distributed in the footpads, scattered in the IFPs. The volar skin is richly innervated. The wound area of the VEWM at 1, 3, 7 and 10 days after the operation is respectively 89.17% ± 2.52%, 71.72% ± 3.79%, 55.09 % ± 4.94% and 35.74% ± 4.05%, and the final scar area accounts for 47.80% ± 6.22% of the initial wound. While the wound area of BEWM at 1, 3, 7 and 10 days after the operation are respectively 61.94% ± 5.34%, 51.26% ± 4.89%, 12.63% ± 2.86% and 6.14% ± 2.84%, and the final scar area accounts for 4.33% ± 2.67% of the initial wound. Fractal analysis of the post-traumatic repair site for VEWM *vs* human was performed: lacunarity values, 0.040 ± 0.012 *vs* 0.038 ± 0.014; fractal dimension values, 1.870 ± 0.237 *vs* 1.903 ± 0.163. Sensory nerve function of normal skin *vs* post-traumatic repair site was assessed: mechanical threshold, 1.05 ± 0.52 *vs* 4.90 g ± 0.80; response rate to pinprick, 100% *vs* 71.67% ± 19.92%, and temperature threshold, 50.34°C ± 3.11°C *vs* 52.13°C ± 3.54°C.

**Conclusions:**

VEWM closely reflects the pathological features of human wound healing and can be applied for skin multiple-appendages regeneration and innervation evaluation.

Highlights

- The volar skin of C57BL/6 mice consists of HFs, SeGs and SwGs, and is richly innervated.
- VEWM closely reflects the pathological features of human wound healing, including filling-based healing of human wounds, the pathological outcomes of human scar formation and sensory function impairment after wounding in humans.
- VEWM can be applied for skin multiple-appendages regeneration and innervation evaluation.

## Background

Skin, the largest organ of the human body with an area of 1.5–2.0 m^2^ and a weight of 3.5–10 kg for an adult, is the interface between the internal organs and the external environment and plays a crucial role in maintaining physiological homeostasis [1]. Human skin tissue has a complex structure comprising the epidermis, dermis and subcutaneous tissue, along with appendages such as hair follicles (HFs), sweat glands (SwGs) and sebaceous glands (SeGs). The skin serves a multitude of diverse functions, as a barrier and defence against foreign agents, and in thermoregulation, lubrication, vitamin production, pigmentation, protection against ultraviolet light, immunological surveillance, sensations of pain and touch, and, most importantly, stem cell niches [2,3]. The skin is highly susceptible to injuries such as burns, trauma, surgical incisions, and metabolic and autoimmune diseases, which result in chronic wounds or scars that not only impair the physiological functions of the skin but can also cause psychological issues and may even lead to death. It has been estimated that burns account for $7.5 billion, chronic wounds for ~$50 billion and scars for nearly $12 billion of healthcare costs each year in the USA [4], while in China, the cost for managing chronic wounds was ~$8010 per capita in 2018 [5].

Skin appendages and nerves are essential to maintain the normal physiological functions of the skin. HFs are involved in physical defence, thermal insulation, social communication, and so on. SwGs primarily secrete water containing electrolytes and play an important role in the regulation of temperature homeostasis and water–salt metabolism balance. SeGs produce sebum that lubricates the skin. Cutaneous innervation involves various sensory and motor nerves. Sensory nerve fibres distributed on the skin can be classified into three groups: (1) unmyelinated C group fibres, responsible for pain, touch and temperature sensation; (2) A-δ fibres involved in the perception of mechanical stimuli and rapid-onset pain; and (3) A-β fibres that respond to light, tactile pressure and proprioception [6]. The skin also has some motoneurons that mainly regulate skin functions such as SwG secretion and vasoconstriction by secreting neurotransmitters, neuromodulators and neuropeptides [7].

Perfect skin regeneration is the holy grail being pursued by researchers and clinicians. Here, ‘perfect’ means not only rapid scar-free healing but also the regeneration of multiple appendages and nerves present in the skin before wounding. Current efforts for regenerating skin appendages and nerves typically involve the use of various types of seeded cells, scaffold materials, bioactive substances and signalling pathways. Advances in technologies such as stem cells, reprogramming, microfluidics, organoids, 3D printing and smart biomaterials have helped in achieving promising results in both *in vivo* and *in vitro* single-appendage and multi-appendage regeneration [8–13]. In addition, our understanding of the molecular mechanisms involved in scar formation and appendage or nerve regeneration during wound healing, such as Cxcl12, engrailed-1 (En1), trps1, CX3CR1, interleukin (IL)-1β and Lef1, has also improved [14–22]. Although at present, autologous skin grafting is considered the gold standard treatment for severe skin wounds, further work on the developments mentioned above may make it possible to regenerate perfect skin in the foreseeable future.

The aim of skin-wound treatment has changed from single-appendage regeneration to the regeneration of multiple appendages and nerves. However, a suitable animal model to evaluate the regeneration effect is yet to be developed. This study aims to develop a volar skin excisional wound model (VEWM) using the C57BL/6 mouse to evaluate the effects of simultaneous regeneration of multiple skin appendages (HFs, SwGs and SeGs) and nerves of the skin. In rodents, wound healing occurs through contraction, which is difficult to simulate in humans, as in the latter, wound healing occurs through a filling process. We hope that the results of the present study may help in providing a solution to this formidable challenge and give rise to a new research paradigm for achieving perfect skin regeneration.

## Methods

### Animals

Non-pregnant female C57BL/6 mice (8–10 weeks old) were purchased from SiBeiFu Bioscience (Beijing). Human scar tissues were obtained from the Department of Burns of the Fourth Medical Center of Chinese PLA General Hospital. Experimental protocols were approved by the Ethics Committee at the Fourth Medical Center of PLA General Hospital and were in compliance with Institutional Animal Care and Use Committee guidelines (approval No. 2019-X15–50). The mice were housed (five per cage) under standard conditions and were provided with rodent water and food. All mice were acclimated to the environment for 1 week before the experiment.

### Well-established protocol for the VEWM

The mice were anesthetized for 10 min in a chamber prefilled with 2.5% isoflurane in 100% oxygen before the surgery was performed. Stable anaesthesia was maintained during the subsequent surgical procedures using 2.0% isoflurane and 40% oxygen in the hand-line nozzle of an anaesthesia unit containing an ether–air mixture (RuiWoDe, China). The volar skin surface of the hindpaw was cleaned and sterilized with povidone-iodine, followed by rinsing with 75% (vol/vol) ethanol. The mouse was placed on a sterile sheet.

The volar skin of the mouse hindpaw was observed to determine the position of the intended wound. Taking the proximal plantar side of the mouse hindpaw as the needle-entry point, a microsyringe (KangDeLai, China) was used to stir up the full-thickness skin and inject 30 μl of normal saline containing thrombin (2 U/ml), factor XIII (10 U/ml) and CaCl_2_ (10 mM) (Sigma). After waiting for 2 min, a hole-punch apparatus (DeLi, China) with an adjustable aperture was used to punch the hindpaw volar skin to determine the location and size of the intended wound. Along the punched boundary, the operator cut off the full-thickness skin within the determined range using microsurgical instruments (ChengHe, China), taking care not to damage the subcutaneous fascia. After making the wound, bleeding was stopped using a sterile gauze or cotton ball for 20 min.

One side of a prefabricated silicone ring was coated with a small amount of biological glue (3 M, USA) and then placed so that it surrounded the wound. The silicone ring was secured with a single suture of 4–0 non-absorbable medical silk thread (JinHuan, China). Next, small-molecule drugs or a hydrogel drug-controlled release system loaded with drugs acting on the wound surface and Tegaderm™ sterile transparent dressing (3 M, USA) were applied to seal the wound. The wound sites were covered using a self-adhering elastic bandage (VetWrap, 3 M, USA).

The mice were placed under a warming lamp until they fully recovered from anaesthesia and were then housed in individual cages in a clean facility. The animals were checked daily to ensure that their bandage was still on.

### Photographing and harvesting wound tissue

The transparent dressing was uncovered and photographs of the individual wounds were taken at appropriate times. At the culmination of the experiment, the mice were sacrificed by isoflurane overdose and cervical dislocation and imaged with a high-resolution digital camera (Canon DS126601, Japan). It is easy to overlook that ruler reference is necessary when taking photographs for subsequent data analysis. The volar skin was cut off with microdissection scissors and unfolded with the epidermis facing up. Then, the specimens were fixed in 4% paraformaldehyde, embedded in paraffin, and cut into 5-μm-thick sections for haematoxylin and eosin (H&E), Masson and immunofluorescence staining.

### Measurement and analysis

The hairs on the volar skin were counted under a microscope. Two investigators performed the procedure separately, and the average value was reported. Wound closure was calculated using the following formula: area of actual wound/area of original wound × 100 [23]. The pixel area was obtained by using Photoshop. Closure fractions were normalized to day 0 for each mouse sample. Investigators were blinded to treatment group identity during the analysis. A H&E staining kit and a Masson staining kit (Solarbio Science & Technology, China) were used for histological analysis according to the manufacturer’s instructions. Fractal analysis was performed using the ImageJ plug in ‘FracLac’, according to the protocol previously described [24].

### Visualization of skin appendages in mouse volar skin

The dissected volar skin from the mouse hindpaw was incubated in Dispase II (Solarbio, China) for 24 h at 4°C, as previously described [25,26]. Next, the epidermis was peeled away from the underlying dermis. Epidermal whole-mount preparations were stained with 0.1% solution of Nile Blue A (Sigma, USA) for 1 min and with 0.5% Oil Red O (Sigma, USA) for 10 min, which stain the eccrine gland ducts and sebaceous glands, respectively. When observed under a stereomicroscope with a camera, the eccrine gland ducts were dyed blue while the HF-associated sebaceous glands were dyed red.

### Immunofluorescence staining

Tissue sections were fixed in 4% paraformaldehyde (PFA) and blocked with phosphate-buffered saline + 0.1% Tween (PBST) + 10% goat serum before incubating in primary antibodies at 4°C overnight and in secondary antibodies at room temperature for 2 h. PBS was used to rinse the sections between steps. The following primary antibodies were used: anti-alpha 1 sodium potassium ATPase (1 : 400, ATP1a1, ab7671, Abcam, USA), anti-LHX2 (1 : 500, ab184337, Abcam, USA) and anti-fatty acid synthase (1:400, FASN, ab128870, Abcam, USA). The secondary antibodies were used: goat antimouse IgG H&L (Alexa Fluor 488) (1 : 200, ab150113, Abcam, USA). The images were captured with a Leica fluorescence microscope.

### RNA extraction and qRT-PCR

At least 10 mice were used. The harvested volar skin or wound samples were collected and washed with PBS, and then the protruding footpads (FPs) or flat inter-footpads (IFPs) were divided with a scalpel. Total RNA was extracted with Trizol (Invitrogen, USA) using the standard protocol. The PrimeScript RT reagent kit (TaKaRa, Japan) was used to synthesis cDNA. qPCR was conducted using SYBR Green Supremix (Bio-Rad, USA) on QuantStudio™ 5 real-time PCR instruments, according to the manufacturer’s instructions. Data were analysed using the 2 − ΔΔCt method. The quantification of target genes was normalized using primers that amplified the β-actin mRNA. The following primer information was used: mouse-β-actin-F, CATGTACGTTGCTATCCAGGC; mouse-β-actin-R, CTCCTTAATGTCACGCACGAT; mouse-En1-F, CTACTCATGGGTTCGGCTAAC; mouse-En1-R, CTTGTCTTCCTTCTCGTTCTTT; mouse-LHX2-F, GAATACCCAGCACACTTTAACC; and mouse-LHX2-R, CATCGTTCTCGTTACAGCTAAG.

### Iodine–starch sweat assay

The iodine–starch sweat assay was conducted by referring to the method described in a previous study [27]. Here, 2% (w/v) iodine/ethanol solution was applied to the volar surface of the mice under anaesthesia. After drying, the surface was coated with 1 g/ml of starch/castor suspension. Then, sweat secretion was stimulated by subcutaneously injecting acetylcholine chloride (2.5 mg/kg). Representative images were taken when the black dots became stable. Two researchers counted the black dots independently under a stereomicroscope and recorded the average value.

### Behavioural response assessment for mouse volar skin perception

#### Temperature sensitivity assay

Paw-withdrawal temperature was determined as described elsewhere [28]. The mice were placed in a Von Frey chamber and acclimated for 30 min. The thermal probe (MouseMet Thermal, Topcat Metrology Ltd, USA) was preheated to 37°C and carefully placed against the intended volar surface of the mouse paw and a force of ~1 g was applied. After contact, the probe was heated at a rate of 2.5°C/s. When the mouse removed its paw from the probe, the temperature on the readout was recorded. Three trials were conducted on each paw and the average value was reported.

#### Mechanical sensitivity assay

The mechanical threshold was defined as the lowest force that resulted in at least three withdrawals in five tests. The mice were acclimated for 30 min in a Von Frey chamber before the test. The mechanical sensitivity threshold was measured with calibrated Von Frey filaments ranging from 0.01 to 6 g. The interval time was at least 30 s between each trial to give the sensory receptors enough time to return to baseline [29].

#### Response to pinprick

The mice were acclimated for 30 min in a Von Frey chamber. A 27-gauge needle was used to stab the intended volar skin of the hindpaw, taking care not to pierce through the skin. Every mouse underwent 10 trials with at least 1-min intervals. A response was manifested as paw withdrawal, shaking or licking and was reported as a percentage of the total number of trials. When mice did not give any response in 20 s, it was defined as no response. The operators were blinded to group information while performing the behavioural experiments.

### Statistical analysis

Data were expressed as mean ± SD and analysed using Graphpad Prism 9.3. The data were checked for normality using the Shapiro–Wilk test. Discrepancy between the two groups was determined using Student’s *t*-test. One-way and two-way ANOVA with multiple comparison tests were performed at multiple time points to compare the data between the two groups.

## Results

### Volar skin of C57BL/6 mice containing HFs, SeGs and SwGs

**Figure 1:**
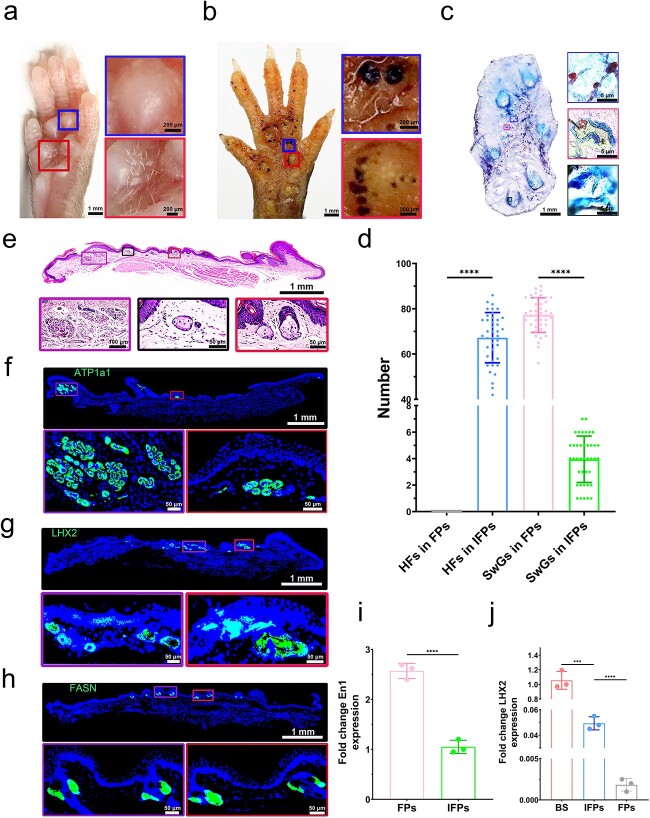
The volar skin of C57BL/6 mice comprises HFs, SeGs and SwGs. (**a**) Gross phenotype of the volar skin of C57BL/6 mouse hindpaw. Hairs are present in IFPs and not on top of the footpads (Scale bar: 1 mm&200 μm). (**b**) Representative images of iodine–starch sweat test on C57BL/6 mouse hindpaw. Sweat spots appear both in IFPs and the footpads (Scale bar: 1 mm&200 μm). (**c**) Representative images of double-staining with Nile Blue A and Oil Red O for the volar epidermal preparation of C57BL/6 mice. Blue SwG ducts stained with Nile Blue A are seen in the FPs or IFPs. Red HF-related SeGs stained with Oil Red O are only seen in IFPs (Scale bar: 1 mm&5 μm). (**d**) Quantification of HFs and SwGs in the footpads and IFPs of C57BL/6 mouse hindpaw (n = 40). (**e**) H&E staining of the volar skin in C57BL/6 mouse hindpaw. The purple boxed area shows SwGs in FPs, the black and red boxed areas show HFs and SeGs in IFPs (Scale bar: 1 mm, 100 μm&5 μm). (**f**–**h**) Single immunofluorescent staining of ATP1a1 (SwGs marker), LHX2 (HF marker) and FASN (SeGs marker) of the volar skin in C57BL/6 mouse (cont.) hindpaw. ATP1a1/LHX2/FASN, green; DAPI, blue. Similarly, the results show that HFs and SeGs are present in IFPs only and SwGs appear in both IFPs and the FPs (Scale bar: 1 mm&50 μm). (**i**, **j**) Differential gene expression of SwGs (En1) and HFs (LHX2) in three tissues of the C57BL/6 mice (FPs, IFPs, BS). For all qRT-PCR analyses, gene expression was normalized to the reference gene (β-actin). The mean and standard deviation ranges are plotted. ^***^p < 0.05, ^****^p < 0.01. *IFPs* inter-footpads, *FPs* footpads, *HFs* hair follicles, *SwGs* sweat glands, *SeGs* sebaceous glands, *BS* back skin, *H&E* haematoxylin and eosin,* ATP1a1* anti-alpha 1 sodium potassium ATPase,* FASN* anti-fatty acid synthase

The gross morphology of the volar skin appendages was examined through macroscopic observation, Oil Red O and Nile Blue A double-staining, and iodine–starch tests. Hair growth can be clearly observed in the volar skin of C57BL/6 mice. However, the hair growth was limited to the IFP area only, and no hair was observed on the FPs (Figure 1a). The iodine–starch test revealed SwGs as black dots. Notably, black dots were seen not only on the two metatarsal, four interdigital and five apical FPs, but also in the areas proximal to the grooves transverse to the axis of each digit and in the areas surrounding or between the interdigital pads (Figure 1b). This result indicates that SwGs were densely distributed in the FPs but scattered in the IFPs of the mice. SwGs and SeGs in the pilosebaceous unit were labelled by Nile Blue A and Oil Red O double-staining (Figure 1c). Based on the data from a sample size of 40 mice, we determined the number of hairs on their volar skin as 67.23 ± 11.09, the number of SwGs in FPs as 77.25 ± 7.64 and the number of SwGs in IFPs as 3.95 ± 1.75 (Figure 1d). The results indicated that the volar skin of C57BL/6 mice contains HFs, SeGs and SwGs.

The micromorphology of the mouse skin was observed through H&E staining. Both HFs and SwGs can be seen in the IFPs, whereas only SwGs were present in the FPs of the mice (Figure 1e). This result can be further confirmed by fluorescent staining: both the SwG-specific marker alpha 1 sodium potassium ATPase (ATP1a1) and the HF-specific marker LHX2 were detected in the IFPs. LHX2 was not found in the FPs (Figure 1f, g). The SeG-specific marker fatty acid synthase (FASN) can only be seen in IFPs, and its expression can be seen close to HFs as well (Figure 1h). Genetic analysis of skin appendages was conducted using qRT-PCR. The expression of the SwG-related gene En1 in the volar skin of the FP was significantly higher than that of the IFPs. In addition, the expression of LHX2 was detected only in IFPs and was lower than that in the back skin (Figure 1i, j).

**Figure 2:**
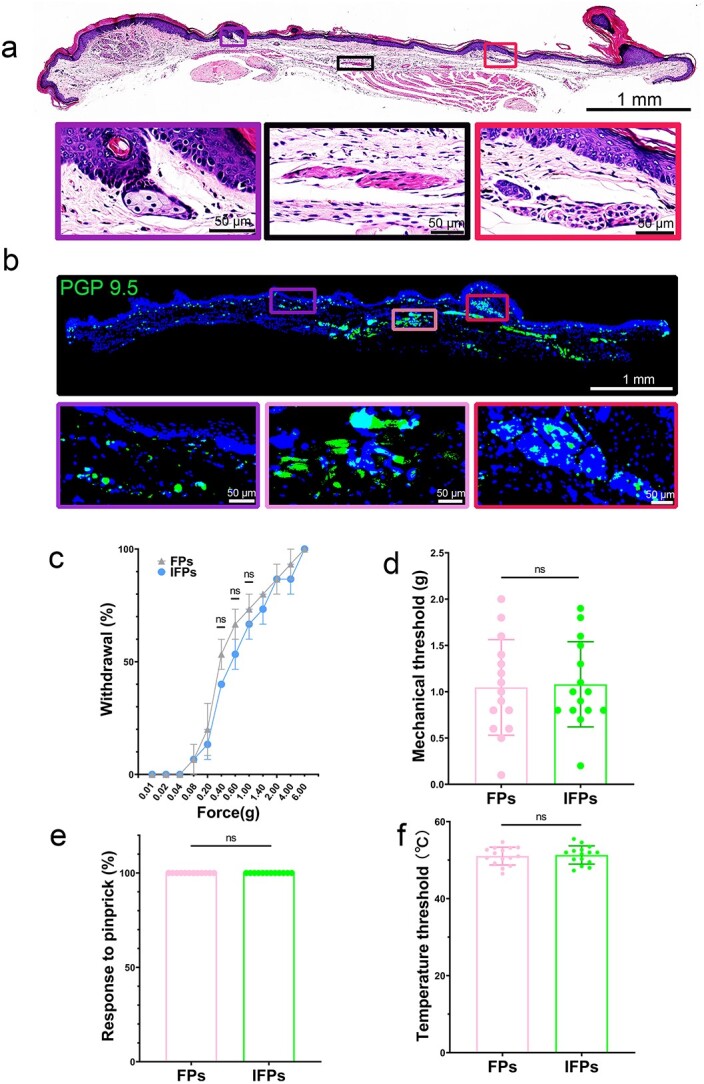
The volar skin of C57BL/6 mice is richly innervated. (**a**) H&E staining of the volar skin in C57BL/6 mouse hindpaw. The purple boxed area shows HFs and SeGs in IFPs, the black boxed area shows muscle tissue in IFPs, and the red boxed area shows SwGs in IFPs (Scale bar: 1 mm&50 μm). (**b**) Single immunofluorescent staining of PGP 9.5 (nerve marker) of the volar skin in C57BL/6 mouse hindpaw. PGP 9.5, green; DAPI, blue. The epidermis, skin appendages (SwGs, HFs, SwGs) and muscle tissue in the volar skin of C57BL/6 mouse hindpaw are all richly innervated (Scale bar: 1 mm&50 μm). (**c**) Percent paw withdrawal (five trials) in response to von Frey filament at different forces in C57BL/6 mice, n = 10. ns, means no statistical significance. (**d**) Mechanical threshold measured in the range 0.01 to 6 g in C57BL/6 mice (n = 15). (**e**) Percent response (three trials) to pinprick on hindpaw in C57BL/6 mice (n = 12). (**f**) Thermal paw withdrawal threshold measured with a thermal probe in C57BL/6 mice (n = 15).* ns* not significant, *IFPs* inter-footpads, *HFs* hair follicles, *SwGs* sweat glands, *SeGs*, sebaceous glands, *H&E *haematoxylin and eosin

To visualize the innervation of the mouse volar skin, anti-PGP 9.5 antibodies were used to label the cutaneous nerve fibres. The superficial nerve plexus runs along the dermis–epidermis junction in the dermis. Small sensory nerve fibres sprout into the epidermis and reach its upper layers (Figure 2a, b). Autonomic nerve fibres are the main fibres that innervate the skin appendages, while motor nerve fibres innervate the skin musculature (Figure 2a, b). Cutaneous nerve fibres are responsible for the perception of a stimulus, such as thermal and tactile sensation or pain, which is recognized and transmitted by different nerve fibres. Behavioural assessments were conducted to obtain the normal response threshold, which can be used as a reference for evaluating cutaneous nerve injury or recovery. The threshold for a temperature response was 51.06°C ± 2.32°C; the mechanical stimulus threshold, used as an innocuous indicator, was 1.05 g ± 0.52 g; and the noxious stimulus index, defined as the response rate to pinprick, was 100%. No significant difference was observed in the temperature threshold, mechanical threshold and response rate to pinprick at two different parts of the FP and IFP (Figure 2c–f).

### Construction of the VEWM

**Figure 3:**
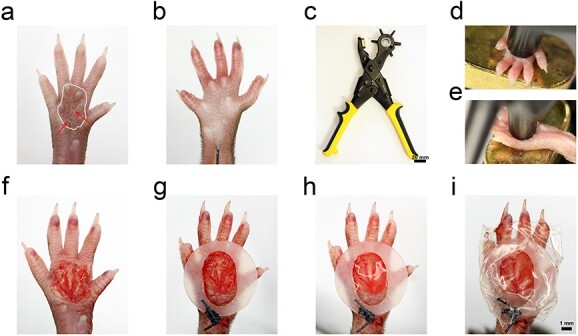
Construction process of the C57BL/6 mice volar skin excisional wound model. (**a**) The position of the intended wound is an ellipse area (white line) covering the four interdigital foot pads and the volar skin in between. (**b**) Physiological saline containing hemostatic ingredients was injected into the subcutaneous tissue in advance to separate the subcutaneous tissue. (**c**–**e**) The hole punch apparatus (DeLi, China) with adjustable aperture was applied to punch the hindpaw volar skin to clarify the location and size of the intended wound (Scale bar: 20 mm). (**f**) The wound made by this method. Well-preserved fascia and some adipose tissue can be seen on the surface. (**g**) A prefabricated oval silicone ring of ~2 x 4 mm was applied to the created wound to provide a defined space for the intervention. (**h**, **i**) Sterile dressing was used to seal the wound, so that the drugs or biological materials loaded into the wound were fixed and protected (Scale bar: 1 mm)

The area of the volar skin of a C57BL/6 mouse is ~2.3 × 4.2 mm^2^. The position of the wound is highly important in this model. Based on the results of a previous study, we suggest that the wound should be located in an ellipse area covering four interdigital FPs and the volar skin between them (Figure 3a).

Surrounding tissues can provide a suitable microenvironment for organ regeneration. The regeneration of skin appendages and nerves is inseparable from the nutritional and structural information provided by the surrounding tissues, including subcutaneous fat, fascia and muscles. Therefore, while making the model, at least the dermis where the appendages and peripheral nerves are located should be removed and the damage to other tissues should be reduced as much as possible. However, the volar skin on the hindpaws of mice is tighter than that on other parts, making it difficult to properly remove the dermis by conventional operation. Therefore, we injected physiological saline containing haemostatic ingredients into the subcutaneous tissue of the mice before separating their subcutaneous tissue (Figure 3b). A hole-punch apparatus with an adjustable aperture was used to punch the hindpaw volar skin to determine the location and size of the intended wound (Figure 3c–e). The wound made by this method preserved the fascia quite well. In addition, some adipose tissue can be seen on the surface, which satisfies the requirement of the resection depth.

Haemostasis affects the loading of drugs or biomaterials on wounds. Although the hindpaw volar skin of mice is rich in blood supply, a good haemostasis effect could be obtained (Figure 3f) when thrombin (2 U/ml), factor XIII (10 U/ml) and CaCl_2_ (10 mM) were used, in conjunction with pressing the wound for a certain period of time. Next, a prefabricated oval silicone ring of ~2 × 4 mm^2^ was applied to the created wound to provide a defined space for the intervention (Figure 3g). Finally, a sterile dressing was used to seal the wound (Figure 3h, i) so that the drugs or biological materials loaded into the wound were fixed and protected. Of course, better protection can be achieved if suitable foot covers are added and anti-bite collars are used.

### VEWM closely reflects pathological features of human wound healing

#### VEWM could mimic filling-based healing of human wounds

**Figure 4:**
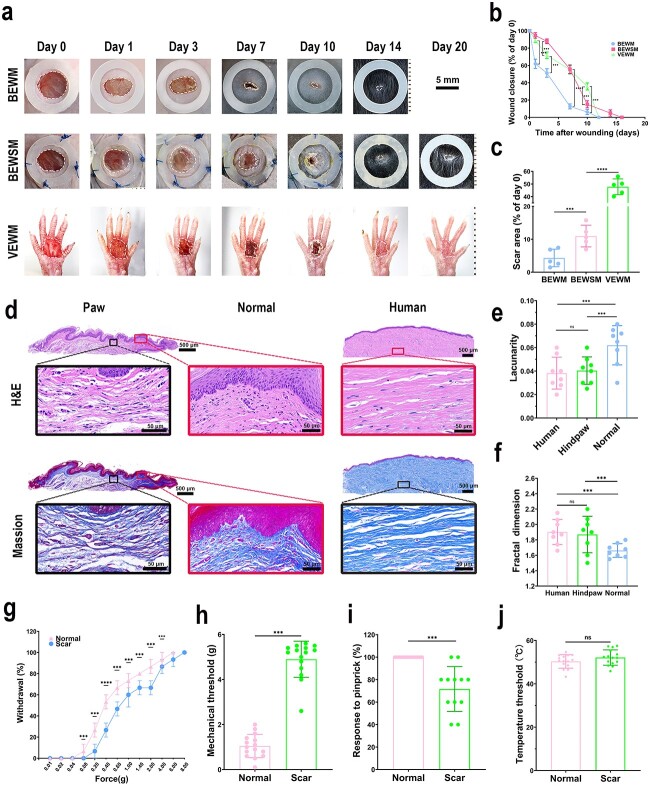
VEWM closely reflects the pathological features of human wound healing. (**a)** Changes in the wound closure areas of different animal models (BEWM, BEWSM, VEWM). The silicone ring of BEWM was placed around the wound to facilitate observation, while the silicone ring of BEWSM was fixed around the wound by suture to prevent contraction (Scale bar: 5 mm). Statistical analysis of the healing of skin defect (**b**) and scar area (**c**); n = at least four wounds. (**d**) H&E and Masson staining of the scar tissue formed after wound healing in C57BL/6 mouse hindpaw and human forearm skin. The middle red box shows normal skin without trauma in the marginal portion of the C57BL/6 mouse hindpaw volar skin (Scale bar: 50 µm&500 µm). Lacunarity values (**e**) and fractal values (**f**) derived from the cyan channel of Masson-stained sections; mean ± SD, n = 8 optical fields. Lacunarity values quantitatively assess the porosity. Fractal dimensions quantitatively assess the complexity. Porous tissue has higher lacunarity values than smoother tissue and complex arrangements have higher fractal score values than simpler ones. (**g–j**) Evaluation of nerve function in normal skin and posttraumatic repaired skin. (g) Percent paw withdrawal (five trials) in response to von Frey filament at different forces in C57BL/6 mice, n = 10. (h) Mechanical threshold measured in the range of 0.01 g to 6 g in C57BL/6 mice (n = 15). (i) Percent response (five trials) to pinprick on hindpaw in C57BL/6 mice (n = 12). (j) Thermal paw withdrawal threshold measured with a thermal probe in C57BL/6 mice (n = 15). The mean ± SD ranges are plotted, ***p < 0.05, ****p < 0.01; ns, not significant. *BEWM* back excisional wound model, *BEWSM* back excisional wound splinting model, *VEWM* volar skin excisional wound model, *H&E* haematoxylin and eosin

Wound closure mainly occurs through contraction in mice, whereas it occurs through filling in humans. As shown by the back excisional wound model (BEWM) in Figure 4a, b, the wound area was 61.94% ± 5.34% on the first day after the wound was created, 12.63% ± 2.86% on the seventh day, and 6.14% ± 2.84% on the tenth day. Putting silicone rings on the edge of the wound can effectively resist the shrinkage. The back excisional wound splinting model (BEWSM) shows that the wound area was almost unchanged on days 1 and 3 after the operation and then reduced to 55.68% ± 4.94% on day 7 (Figure 4a, b). Fresh granulation tissue can be seen on the wound base and edge, indicating that filling-based wound healing in humans can be mimicked by inhibiting contraction in mice. However, the BEWSM cannot completely eliminate the influence of the intrinsic contractile potential on the healing process in mice. The final scar area in the BEWM accounts for 4.33% ± 2.67% of the original wound area, compared with 10.97% ± 3.30% for the BEWSM (Figure 4c). In addition to the influence of the mouse’s own hair cycle (17–19 days), it is difficult for researchers to identify the location of the initial wound, which also makes subsequent tissue-level detection inconvenient. The VEWM will alleviate the abovementioned challenges. The wound areas of the VEWM on days 1, 3, 7 and 10 after operation are 89.17% ± 2.52%, 71.72% ± 3.79%, 55.09% ± 4.94% and 35.74% ± 4.05%, respectively. Compared with the other two models (BEWM and BEWSM), the VEWM shows signs of filling-based healing, and the curve is smoother than that achieved using the other two models (Figure 4a, b). The final scar area accounts for 47.80% ± 6.22% of the initial wound, which is significantly higher than that of the other two models (Figure 4c). Hence, the VEWM can simulate the filling-based healing of human wounds. Moreover, the final scar area remains stable. No significant reduction in the scar area was observed 30 days after operation. In addition, the problem that the wound site is easily influenced by its own hair cycle is avoided.

#### VEWM could mimic the pathological outcomes of scar formation in humans

We conducted H&E and Masson staining to verify whether the VEWM could mimic the pathological outcomes of scar formation in humans. In normal skin, collagen was arranged in a basket-weave pattern, whereas the fibres in scar collagen were arranged in parallel (Figure 4d). Fractal analysis indicated that lattice arrangement at the injury site was not different between the VEWM scar tissues in humans and C57BL/6 mice: lacunarity values 0.040 ± 0.012 *vs* 0.038 ± 0.014 and fractal dimension values 1.870 ± 0.237 *vs* 1.903 ± 0.163 (Figure 4e, f). The lacunarity values were used to quantitatively assess porosity, while the fractal dimensions were used to quantitatively assess complexity. The lacunarity values of the porous tissue were higher than those of smoother tissues. The complex arrangements of scar tissues have higher fractal dimension values than those of the simpler arrangements.

#### VEWM could mimic sensory function impairment after wounding in humans

A behavioural response assessment was performed to demonstrate that the VEWM could mimic the sensory function impairment occurring after a wound in humans. Compared with the normal volar skin, the post-traumatic repair site showed obvious symptoms of sensory nerve-function damage: the withdrawal rate of mechanical stimulation decreases (Figure 4g), the threshold of mechanical stimulation increases (1.05 *g* ± 0.52* g*  *vs* 4.90 *g* ± 0.80* g*) (Figure 4h) and the response to pinprick rate decreases (100% *vs* 71.67% ± 19.92%) (Figure 4i). Notably, the temperature threshold is not significantly different from that of the normal volar skin (50.34°C ± 3.11°C *vs* 52.13°C ± 3.54°C) (Figure 4j).

## Discussion

BEWMs of mice are widely used as a mature animal model for evaluating hair follicle regeneration [11,30–34]. SeGs often depend on HFs to exist. Therefore, this model can also be used to evaluate the regeneration of SeGs [35,36]. However, this rodent-based model has a drawback too: the rodents in which most research about wound healing and regeneration has been performed differ from humans in important ways. Their skin is loose, whereas that of humans is tight. Their wounds heal by contraction, i.e. such wounds pull together rather than filling in [19,37]; therefore, it becomes necessary to use an anti-constriction ring (BEWSM). However, as the volar skin of rodents is relatively dense and it does not show any obvious contraction behaviour during the wound-healing process, an anti-contraction ring is not needed, which is why we chose the volar skin to establish our model.

Unfortunately, the BEWSM cannot be used to study SwGs. Previous studies have demonstrated that SwGs are restricted to the volar skin in mice and rats [26,38]. It is difficult to regenerate SwGs at a site that does not have SwGs itself. The hindpaw scald model of mice is the most commonly used one for assessing SwG regeneration [39–42] and is simple to operate. Although scalding inactivates the epidermis of the FP, it retains its structural integrity. As a result, it remains conducive to the attachment and protection of intervening components such as cells and can also prevent infection. Nevertheless, several limitations of the scald model have been reported. Firstly, it is difficult to control the degree of scalding of the FP. Ideally, a full-thickness scald model with complete destruction of SwGs and minimal damage to the subcutaneous tissue and surrounding environment is expected. Our research has shown that when the heat is insufficient, some SwGs will remain. Consequently, it becomes difficult to determine whether the restored SwGs are regenerated under the influence of intervention measures or are a result of the repair of the residual SwGs. For now, it is difficult to answer this question for regenerative-capacity assessment. Previous studies based on this model have also raised several questions [43]. When the heat is excessive, the damage is massive and will lead to loss of the tissue environment that can support regeneration. In extreme cases, the entire paw of the mouse may become necrotic and fall off, making it almost impossible to obtain a qualified full-thickness, third-degree scald model that can be used for the evaluation of SwG regeneration. The description of temperature applied during modelling in the literature is not very clear and ranges from 65°C for 5 s to 65°C for 15 s, or full-thickness third-degree burns, or second-degree burns [39–42]. In addition, the paw-scald model cannot simulate most clinical and research scenarios apart from scalds, such as skin defects caused by trauma, burn wounds after debridement and tissue engineering skin evaluation. Some studies have used a mouse volar trauma model to assess the regeneration of SwGs [44,45]. Although these studies did help in the overall understanding of the phenomenon, the lack of specific details such as wound location, depth, haemostasis, anti-infection and intervention load, limit its wide application.

Our study results confirmed that the volar skin of C57BL/6 mice comprises HFs, SeGs and SwGs and is richly innervated. The VEWM could mimic the pathological progress and outcomes of scar formation and sensory function impairment after wounding in humans, which lays a theoretical foundation for the establishment of an animal model for perfect skin regeneration after injury. The new VEWM not only allows simultaneous evaluation of multiple appendages and nerve regeneration but also addresses the major defect of the volar scald wound mode, i.e. the presence of SwG residues, while minimizing damage to the surrounding tissue environment. Moreover, our research describes the model construction process and index quantification method in detail to devise a protocol that is beneficial for the standardization and wide application of the model.

Loss of sensory function in scars after burn is common. The evaluation of skin sensory nerve regeneration has been carried out on the BEWM of mice [46,47], and the A-β, A-δ and C type fibres are tested by using neurometres, instruments to release electrical stimulations of different frequencies (2000, 250 and 5 Hz, respectively), using two self-adhesive electrodes for linking the wound and the tail [46]. This quantification method is poorly sensitive because of the long distance between the wound and the tail, resulting in a high rate of false negatives. Our research group used the functional recovery behavioural evaluation method of the classical model sciatic nerve injury for evaluating skin wound sensory nerve regeneration. We replaced the intermediate index with the endpoint index, which made the evaluation more scientific and reliable. The values of mechanical stimulus threshold and response rate to pinprick are sometimes contrary to the results reported above. This is because the symptoms of chronic sensory disability following a deep-skin wound not only cause sensibility losses, but also cause itching, paraesthesia and mechanical allodynia [48]. Our data show that the temperature sense is the fastest to recover after a deep-skin wound, which is consistent with reports in other literature [49]. This phenomenon needs to be investigated further.

**Figure 5:**
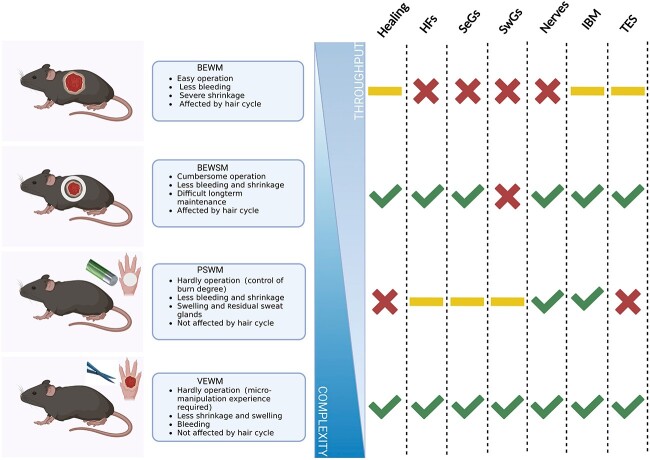
Summary of the characteristics and evaluation of the application of the various models. A yellow line indicates that the model can be used but has significant disadvantages, a green tick indicates that it can be applied, while a red cross indicates that it cannot be applied. *BEWM* back excisional wound model, *BEWSM* back excisional wound splinting model, *PSWM* paw skin scald wound model, *VEWM* volar skin excisional wound model, *HFs* hair follicles, *SwGs* sweat glands, *SeGs* sebaceous glands, *IBM* injectable biomaterials, *TES* tissue-engineered skin

The VEWM can be used under various research scenarios related to skin-regeneration assessment. The characteristics and application scenarios of various models are summarized in Figure 5. Note that our model was established using the hindpaw of C57BL/6 mice. It is not recommended to generalize it to the forepaws of mice and rats because of the differences in the physiological structure of the volar skin of the forepaw and hindpaw, as well as that between mice and rats [50].

Our new model has several limitations also. Firstly, the volar area of mice is limited and the diameter of the wound surface of the model is ~3 mm. In addition, it is necessary to protect the surrounding tissue from unnecessary damage as much as possible. Hence, some experience in microsurgery may be required during modelling. It cannot be used for engineering large volumes of skin tissues; if this is required, large animals such as pigs may be an alternative. Moreover, the volar skin of the mouse is rich in blood supply. Therefore, it becomes essential to quickly achieve effective haemostasis. Finally, despite these limitations, the hindpaw volar scald model is still the best choice for research on burns.

## Conclusions

The volar skin of C57BL/6 mice consists of HFs, SeGs and SwGs, and is richly innervated. VEWM closely reflects the pathological features of human wound-healing and can be applied for skin multiple-appendages regeneration and innervation evaluation.

## Abbreviations

BEWM: Back excisional wound model; BEWSM: Back excisional wound splinting model; BS: Back skin; FPs: footpads; HFs: Hair follicles; IBM: Injectable biomaterials; IFPs: Inter-footpads; PSWM: Paw skin scald wound model; SeGs: Sebaceous glands; SwGs: sweat glands; VEWM: Volar skin excisional wound model; TES: Tissue-engineered skin.

## Funding

This work was supported in part by the National Nature Science Foundation of China [92268206, 81830064]; the CAMS Innovation Fund for Medical Sciences [CIFMS, 2019-I2M-5-059]; the Military Medical Research Projects [145AKJ260015000X; 2022-JCJQ-ZB-09600, 2020-JCJQ-ZD-256-021]; the Military Medical Research and Development Projects [AWS17J005, 2019-126]; and the Specific Research Fund of The Innovation Platform for Academicians of Hainan Province [YSPTZX202317].

## Authors’ contributions

XY Sun, XB Fu, AZ Cai designed the trial and control the supervised experiment implementation. HH Gao, YQ Liu, HL Zhang, MY Wang, LX Zhou, YY Hong and L Wu participated in relevant animal experiments and obtained specimens. ZW Shi, HT Chen, Y Li, SF Ji, JB Xiang and W Pi participated in statistical analysis. HH Gao, YQ Liu and ZW Shi drafted the manuscript. All authors discussed the results and contributed to manuscript revision.

## Ethics approval

Experiment protocols were approved by the Ethics Committee at the Fourth Medical Center of PLA General Hospital and in compliance with Institutional Animal Care and Use Committee guidelines (approval No. 2019-X15–50).

## Conflict of interests

None declared.

## Data availability

All data are presented in the main manuscript.

## References

[1] Jung EG, Augustin M. Dermatologie. Georg Thieme Verlag, 2005. https://books.google.com.hk/books?id=9CwPDA2MXOgC.

[2] Woodby B, Penta K, Pecorelli A, Lila MA, Valacchi G. Skin health from the inside out. Annu Rev Food Sci Technol. 2020;11:235–54. 10.1146/annurev-food-032519-051722. 31905017

[3] Gravitz L . Skin. Nature. 2018;563:S83. 10.1038/d41586-018-07428-4. 30464282

[4] Rodrigues M, Kosaric N, Bonham CA, Gurtner GC. Wound healing: a cellular perspective. Physiol Rev. 2019;99:665–706. 10.1152/physrev.00067.2017. 30475656 PMC6442927

[5] Fu X . State policy for managing chronic skin wounds in China. Wound Repair Regen. 2020;28:576–7. 10.1111/wrr.12808. 32185853

[6] Horch KW, Tuckett RP, Burgess PR. A key to the classification of cutaneous mechanoreceptors. J Invest Dermatol. 1977;69:75–82. 10.1111/1523-1747.ep12497887. 874346

[7] Björklund H, Dalsgaard CJ, Jonsson CE, Hermansson A. Sensory and autonomic innervation of non-hairy and hairy human skin. An immunohistochemical study. Cell Tissue Res. 1986;243:51–7. 10.1007/bf00221851. 2417723

[8] Zhang Y, Enhejirigala, Yao B, Li Z, Song W, Li J, et al. Using bioprinting and spheroid culture to create a skin model with sweat glands and hair follicles. Burns Trauma. 2021;9:tkab013. 10.1093/burnst/tkab013. 34213515 PMC8240535

[9] Yao C, Zhou Y, Wang H, Deng F, Chen Y, Zhu X, et al. Adipose-derived stem cells alleviate radiation-induced dermatitis by suppressing apoptosis and downregulating cathepsin F expression. Stem Cell Res Ther. 2021;12:447. 10.1186/s13287-021-02516-1. 34372921 PMC8351374

[10] Sun X, Xiang J, Chen R, Geng Z, Wang L, Liu Y, et al. Sweat gland organoids originating from reprogrammed epidermal keratinocytes functionally recapitulated damaged skin. Adv Sci (Weinh). 2021;8:e2103079. 10.1002/advs.202103079. 34569165 PMC8596119

[11] Lee J, Rabbani CC, Gao H, Steinhart MR, Woodruff BM, Pflum ZE, et al. Hair-bearing human skin generated entirely from pluripotent stem cells. Nature. 2020;582:399–404. 10.1038/s41586-020-2352-3. 32494013 PMC7593871

[12] Qi C, Xu L, Deng Y, Wang G, Wang Z, Wang L. Sericin hydrogels promote skin wound healing with effective regeneration of hair follicles and sebaceous glands after complete loss of epidermis and dermis. Biomater Sci. 2018;6:2859–70. 10.1039/c8bm00934a. 30259043

[13] Shen K, Wang XJ, Liu KT, Li SH, Li J, Zhang JX, et al. Effects of exosomes from human adipose-derived mesenchymal stem cells on inflammatory response of mouse RAW264.7 cells and wound healing of full-thickness skin defects in mice. Zhonghua Shao Shang Za Zhi. 2022;38:215–26. 10.3760/cma.j.cn501120-20201116-00477. 35325966 PMC11705281

[14] Mascharak S, Talbott HE, Januszyk M, Griffin M, Chen K, Davitt MF, et al. Multi-omic analysis reveals divergent molecular events in scarring and regenerative wound healing. Cell Stem Cell. 2022;29:315–327.e6. 10.1016/j.stem.2021.12.011. 35077667 PMC8988390

[15] Wang G, Sweren E, Liu H, Wier E, Alphonse MP, Chen R, et al. Bacteria induce skin regeneration via IL-1β signaling. Cell Host Microbe. 2021;29:777–91.e6. 10.1016/j.chom.2021.03.003. 33798492 PMC8122070

[16] Mascharak S, des Jardins-Park HE, Davitt MF, Griffin M, Borrelli MR, Moore AL, et al. Preventing Engrailed-1 activation in fibroblasts yields wound regeneration without scarring. Science. 2021;372:356–62. 10.1126/science.aba2374. PMC9008875 33888614

[17] Phan QM, Fine GM, Salz L, Herrera GG, Wildman B, Driskell IM, et al. Lef1 expression in fibroblasts maintains developmental potential in adult skin to regenerate wounds. elife. 2020;9:1–19. 10.7554/eLife.60066. PMC7524549 32990218

[18] Kolter J, Feuerstein R, Zeis P, Hagemeyer N, Paterson N, d'Errico P, et al. A subset of skin macrophages contributes to the surveillance and regeneration of local nerves. Immunity. 2019;50:1482–97.e7. 10.1016/j.immuni.2019.05.009. 31201094

[19] Willyard C . Unlocking the secrets of scar-free skin healing. Nature. 2018;563:S86–s8. 10.1038/d41586-018-07430-w. 30464288

[20] Nishiguchi MA, Spencer CA, Leung DH, Leung TH. Aging suppresses skin-derived circulating SDF1 to promote full-thickness tissue regeneration. Cell Rep. 2018;24:3383–92.e5. 10.1016/j.celrep.2018.08.054. 30257200 PMC6261459

[21] Rinkevich Y, Walmsley GG, Hu MS, Maan ZN, Newman AM, Drukker M, et al. Identification and isolation of a dermal lineage with intrinsic fibrogenic potential. Science. 2015;348:aaa2151. 10.1126/science.aaa2151. 25883361 PMC5088503

[22] Yuan X, Duan X, Li Z, Yao B, Enhejirigala, Song W, et al. Collagen triple helix repeat containing-1 promotes functional recovery of sweat glands by inducing adjacent microvascular network reconstruction in vivo. Burns Trauma. 2022;10:tkac035. 10.1093/burnst/tkac035. 35937591 PMC9346565

[23] Wang X, Ge J, Tredget EE, Wu Y. The mouse excisional wound splinting model, including applications for stem cell transplantation. Nat Protoc. 2013;8:302–9. 10.1038/nprot.2013.002. 23329003

[24] Jiang D, Correa-Gallegos D, Christ S, Stefanska A, Liu J, Ramesh P, et al. Two succeeding fibroblastic lineages drive dermal development and the transition from regeneration to scarring. Nat Cell Biol. 2018;20:422–31. 10.1038/s41556-018-0073-8. 29593327

[25] Aldea D, Atsuta Y, Kokalari B, Schaffner SF, Prasasya RD, Aharoni A, et al. Repeated mutation of a developmental enhancer contributed to human thermoregulatory evolution. Proc Natl Acad Sci U S A. 2021;118:1–10. 10.1073/pnas.2021722118. PMC8072367 33850016

[26] Kamberov YG, Wang S, Tan J, Gerbault P, Wark A, Tan L, et al. Modeling recent human evolution in mice by expression of a selected EDAR variant. Cell. 2013;152:691–702. 10.1016/j.cell.2013.01.016. 23415220 PMC3575602

[27] Wada M, Takagaki T. A simple and accurate method for detecting the secretion of sweat. Tohoku J Exp Med. 1948;49:284.

[28] Deuis JR, Vetter I. The thermal probe test: a novel behavioral assay to quantify thermal paw withdrawal thresholds in mice. Temperature (Austin). 2016;3:199–207. 10.1080/23328940.2016.1157668. 27857950 PMC4965000

[29] Gladman SJ, Huang W, Lim SN, Dyall SC, Boddy S, Kang JX, et al. Improved outcome after peripheral nerve injury in mice with increased levels of endogenous ω-3 polyunsaturated fatty acids. J Neurosci. 2012;32:563–71. 10.1523/jneurosci.3371-11.2012. 22238091 PMC6621061

[30] Nanmo A, Yan L, Asaba T, Wan L, Kageyama T, Fukuda J. Bioprinting of hair follicle germs for hair regenerative medicine. Acta Biomater. 2022;1–10. 10.1016/j.actbio.2022.06.021. 35718100

[31] Chen P, Miao Y, Zhang F, Fan Z, Huang J, Mao X, et al. Tissue engineering ECM-enriched controllable vascularized human microtissue for hair regenerative medicine using a biomimetic developmental approach. J Adv Res. 2022;38:77–89. 10.1016/j.jare.2021.09.010. 35572404 PMC9091751

[32] Hu S, Li Z, Lutz H, Huang K, Su T, Cores J, et al. Dermal exosomes containing miR-218-5p promote hair regeneration by regulating β-catenin signaling. Sci Adv. 2020;6:eaba1685. 10.1126/sciadv.aba1685. 32832660 PMC7439409

[33] Abaci HE, Coffman A, Doucet Y, Chen J, Jacków J, Wang E, et al. Tissue engineering of human hair follicles using a biomimetic developmental approach. Nat Commun. 2018;9:5301. 10.1038/s41467-018-07579-y. 30546011 PMC6294003

[34] Snippert HJ, Haegebarth A, Kasper M, Jaks V, van Es JH, Barker N, et al. Lgr6 marks stem cells in the hair follicle that generate all cell lineages of the skin. Science. 2010;327:1385–9. 10.1126/science.1184733. 20223988

[35] Nowak JA, Polak L, Pasolli HA, Fuchs E. Hair follicle stem cells are specified and function in early skin morphogenesis. Cell Stem Cell. 2008;3:33–43. 10.1016/j.stem.2008.05.009. 18593557 PMC2877596

[36] Horsley V, O'Carroll D, Tooze R, Ohinata Y, Saitou M, Obukhanych T, et al. Blimp1 defines a progenitor population that governs cellular input to the sebaceous gland. Cell. 2006;126:597–609. 10.1016/j.cell.2006.06.048. 16901790 PMC2424190

[37] Eming SA, Martin P, Tomic-Canic M. Wound repair and regeneration: mechanisms, signaling, and translation. Sci Transl Med. 2014;6:265sr6. 10.1126/scitranslmed.3009337. 25473038 PMC4973620

[38] Coulson-Thomas VJ, Gesteira TF, Esko J, Kao W. Heparan sulfate regulates hair follicle and sebaceous gland morphogenesis and homeostasis. J Biol Chem. 2014;289:25211–26. 10.1074/jbc.M114.572511. 25053416 PMC4155684

[39] Xu Y, Hong Y, Xu M, Ma K, Fu X, Zhang M, et al. Role of keratinocyte growth factor in the differentiation of sweat gland-like cells from human umbilical cord-derived mesenchymal stem cells. Stem Cells Transl Med. 2016;5:106–16. 10.5966/sctm.2015-0081. 26574554 PMC4704873

[40] Huang S, Yao B, Xie J, Fu X. 3D bioprinted extracellular matrix mimics facilitate directed differentiation of epithelial progenitors for sweat gland regeneration. Acta Biomater. 2016;32:170–7. 10.1016/j.actbio.2015.12.039. 26747979

[41] Cai S, Pan Y, Han B, Sun TZ, Sheng ZY, Fu XB. Transplantation of human bone marrow-derived mesenchymal stem cells transfected with ectodysplasin for regeneration of sweat glands. Chin Med J. 2011;124:2260–8. 21933554

[42] Sheng Z, Fu X, Cai S, Lei Y, Sun T, Bai X, et al. Regeneration of functional sweat gland-like structures by transplanted differentiated bone marrow mesenchymal stem cells. Wound Repair Regen. 2009;17:427–35. 10.1111/j.1524-475X.2009.00474.x. 19660052

[43] Bovell DL . The evolution of eccrine sweat gland research towards developing a model for human sweat gland function. Exp Dermatol. 2018;27:544–50. 10.1111/exd.13556. 29626846

[44] Huang S, Lu G, Wu Y, Jirigala E, Xu Y, Ma K, et al. Mesenchymal stem cells delivered in a microsphere-based engineered skin contribute to cutaneous wound healing and sweat gland repair. J Dermatol Sci. 2012;66:29–36. 10.1016/j.jdermsci.2012.02.002. 22398148

[45] Huang S, Xu Y, Wu C, Sha D, Fu X. In vitro constitution and in vivo implantation of engineered skin constructs with sweat glands. Biomaterials. 2010;31:5520–5. 10.1016/j.biomaterials.2010.03.060. 20398932

[46] Blais M, Grenier M, Berthod F. Improvement of nerve regeneration in tissue-engineered skin enriched with schwann cells. J Invest Dermatol. 2009;129:2895–900. 10.1038/jid.2009.159. 19587695

[47] Caissie R, Gingras M, Champigny MF, Berthod F. In vivo enhancement of sensory perception recovery in a tissue-engineered skin enriched with laminin. Biomaterials. 2006;27:2988–93. 10.1016/j.biomaterials.2006.01.014. 16448695

[48] Girard D, Laverdet B, Buhe V, Trouillas M, Ghazi K, Alexaline MM, et al. Biotechnological Management of Skin Burn Injuries: challenges and perspectives in wound healing and sensory recovery. Tissue Eng Part B Rev. 2017;23:59–82. 10.1089/ten.TEB.2016.0195. 27609352

[49] Murthy SE, Loud MC, Daou I, Marshall KL, Schwaller F, Kühnemund J, et al. The mechanosensitive ion channel Piezo2 mediates sensitivity to mechanical pain in mice. Sci Transl Med. 2018;10:1–22. 10.1126/scitranslmed.aat9897. PMC6709986 30305457

[50] Chen Z, Zhao J, Yan Y, Zhang L, Du L, Liu X, et al. Differential distribution and genetic determination of eccrine sweat glands and hair follicles in the volar skin of C57BL/6 mice and SD rats. BMC Vet Res. 2022;18:316. 10.1186/s12917-022-03416-z. 35974330 PMC9380334

